# A Metabolomics-Based Toolbox to Assess and Compare the Metabolic Potential of Unexplored, Difficult-to-Grow Bacteria

**DOI:** 10.3390/md20110713

**Published:** 2022-11-14

**Authors:** Federica Fiorini, Felizitas Bajerski, Olga Jeske, Cendrella Lepleux, Jörg Overmann, Mark Brönstrup

**Affiliations:** 1 Department of Chemical Biology, Helmholtz Centre for Infection Research, 38124 Braunschweig, Germany; 2 Leibniz Institute DSMZ-German Collection of Microorganisms and Cell Cultures (DSMZ), 38124 Braunschweig, Germany; 3 Department of Microbiology, Braunschweig University of Technology, 38124 Braunschweig, Germany; 4 German Center of Infection Research (DZIF), Site Hannover–Braunschweig, 38124 Braunschweig, Germany; 5 Biomolecular Drug Research Center (BMWZ), Leibniz University Hannover, 30167 Hannover, Germany

**Keywords:** LC-MS/MS, untargeted metabolomics, natural products, difficult-to-grow bacteria, novel bacterial strains

## Abstract

Novel high-throughput cultivation techniques create a demand to pre-select strains for in-depth follow-up studies. We report a workflow to identify promising producers of novel natural products by systematically characterizing their metabolomes. For this purpose, 60 strains from four phyla (Proteobacteria, Bacteroidetes, Actinobacteria and Firmicutes) comprising 16 novel species and six novel genera were cultivated from marine and terrestrial sources. Their cellular metabolomes were recorded by LC-MS/MS; data analysis comprised databases MS/MS matching, in silico compound assignment, and GNPS-based molecular networking. Overall, 1052 different molecules were identified from 6418 features, among them were unusual metabolites such as 4-methoxychalcone. Only a minor portion of the 755 features were found in all phyla, while the majority occurred in a single phylogroup or even in a single strain. Metabolomic methods enabled the recognition of highly talented strains such as AEG42_45, which had 107 unique features, among which a family of 28 potentially novel and related compounds according to MS/MS similarities. In summary, we propose that high-throughput cultivation and isolation of bacteria in combination with the presented systematic and unbiased metabolome analysis workflow is a promising approach to capture and assess the enormous metabolic potential of previously uncultured bacteria.

## 1. Introduction

Thanks to their exceptional bioactive properties, natural products (NPs) play a central role in biomedical research. Discovering natural products and deciphering their function not only improves our understanding of microbial ecology but has also led to the development of therapeutic drugs [1,2,3,4]. However, directing research efforts to novel chemical substances and limiting the unproductive and time-consuming isolation of known chemical entities constitutes a major challenge for natural product discovery [4]. 

The chance to find chemical novelty is particularly high from understudied taxa [5,6]. This long-prevailing ‘common sense’ has been substantiated by recent studies. For example, Hoffmann et al. compared the metabolomes of 2300 different myxobacterial strains from 14 genera to their phylogenic diversity and demonstrated a correlation between taxonomic distance and the production of distinct secondary metabolite families [7]. Similar conclusions were drawn from a study of 72 isolates belonging to the actinomycete genus *Planomonospora*, that has shown a correlation between chemical diversity and strain phylogeny using a pipeline of freely available tools for metabolome and genome mining [8]. 

About 70% of the Earth’s surface is covered with water and the oceans hold over 95% of all waters, but only about 20% of environmental bacterial isolates come from aquatic environments, and only half of them originate from marine sediment [9]. Thus, the ocean may contribute a large number of species to the Earth’s bacterial community [10] and genera such as *Salinospora* have already proven to be a prolific source of structurally unique bioactive compounds [11,12].

Liquid chromatography coupled to (tandem) mass spectrometry (LC-MS(/MS)) has become a key technology in natural product dereplication, defined as the process of recognizing previously known substances present in an extract [13,14]. Methods of untargeted metabolomics, originally developed and applied for studying primary metabolism across a broad range of concentrations, have been recently adopted to natural product research in order to capture secondary metabolism from a large number of samples at hitherto unprecedented depth [15]. The change in dereplication has been fueled by coupling high-resolution LC-MS/MS to global databases [16] and novel bioinformatics methods such as SIRIUS4 [17] or Global Natural Products Social Molecular Networking (GNPS) [18] Besides early dereplication, the identification and prioritization of “talented” producer strains out of larger bacterial collections provides guidance to potentially novel chemistries [12,19,20].

Even if untargeted metabolomic profiling is a promising approach, the large amount and high complexity of signals remains a challenge for high-throughput data processing and interpretation [21]. Many novel open software, approaches, and algorithms are constantly developed for the different steps of metabolomic signal processing and analysis [22]. MetaboAnalyst [23] and Workflow4Metabolomics [24], for example, provide different options for data processing and other tools as user-friendly web platforms, but they are focused in particular on the statistical tasks, such as supervised data projection, and dimensionality reduction techniques. Some of these tools cover most of the processing and analysis steps [23,24,25]; however, to the best of our knowledge, there is still no suitable unified procedure available from signal acquisition to data interpretation, which includes dereplication and prioritization of strains for follow-up studies. Thus, our objective was to combine standardized LC-MS/MS measurements with selected open-access metabolomics tools, to provide a comprehensive workflow from signal acquisition to “talented” strain prioritization for guiding the discovery of novel NPs.

With these premises, we studied the metabolic capability of difficult-to-cultivate bacteria, and in particular of novel bacterial species from environmental samples. We report an unbiased endo-metabolomics investigation of 60 crude extracts from novel bacterial strains isolated from marine (water, sediment, algae, sponges) and from terrestrial (soil) sources of different geographical locations. In particular, the study addresses the following aspects: (i) What proportion of metabolite features extracted from a novel bacterial species can be readily assigned to known metabolites, and what proportion is potentially new? (ii) Is there an overlap of unassigned features, or are they unique to a specific strain? (iii) Can a standardized workflow of LC-MS measurements be combined with open-access analysis tools to guide strain selection across phyla to the most talented producers and novel NPs?

## 2. Results and Discussion

### 2.1. Samples Sources, Isolation and Cultivation

**Figure 1:**
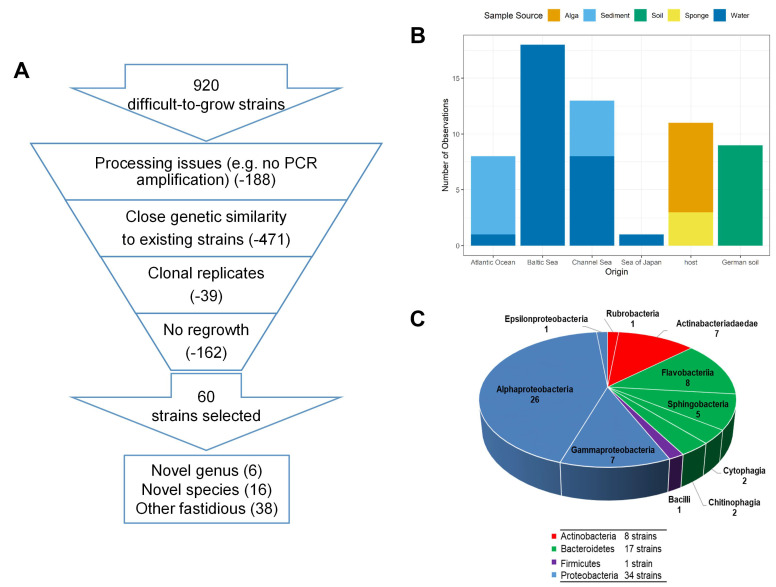
Characteristics of microbial strains. (**A**): Selection cascade from 920 difficult-to-grow strains, cultivated as outlined in Supplementary Figure S1, to the 60 strains investigated in this study. (**B**): Sample source and origin of the selected 60 difficult-to-grow bacterial strains. Samples were collected from four different aquatic marine sources (blue), two aquatic hosts (yellow, orange) and one terrestrial environment (green). (**C**): Pie chart with phylogenetic composition of the 60 strains and number of strains per phylum (blue for Proteobacteria, purple for Firmicutes, green for Bacteroidetes and red for Actinobacteria).

The microbial resource selection focused on difficult-to-grow bacterial strains that were rarely isolated because of their particular growth requirements. Marine samples (water, sediment, algae, sponges) from diverse regional origins, including the Atlantic Ocean, Baltic Sea, Channel Sea and Sea of Japan, and terrestrial samples from German soils, were used in a high-throughput approach that comprised a combination of biofilm, chemotaxis, direct plating and multiwell plate cultivation (Supplementary Figure S1). The combination of broad (high-throughput, different media and cultivation techniques) and selective (longer growth/incubation period for more than two to three days, ambient temperatures, and selective substrates) cultivation approaches aimed at the isolation of those microorganism that usually would not grow using standard cultivation conditions or that would be easily overgrown by so-called fast-growers. Thus, all our isolates are supposed to be slow growing fastidious bacteria. Starting from a pool of more than 900 strains of 264 different species, a set of 60 novel (based on 16S rRNA nucleotide sequence similarity to described species) and/or fastidious (based on selective isolation) marine isolates as well as some difficult-to-grow terrestrial soil isolates were selected (Figure 1A,B). Strains that did not regrow, failed in analyses or were potential clonal replicates were removed from further analysis. Using the 95% [26] and 98.7% [27] threshold values currently recommended to determine the affiliation of bacterial isolates to an existing or new genus or species, six novel genera and 16 novel species were identified. The isolated bacteria could be assigned to the phyla Proteobacteria (35), Bacteroidetes (17), Actinobacteria (8) and Firmicutes (1), thereby representing the dominant marine (here, with except of the soil isolates) bacterial community known from the literature and databases such as Bac*Dive* (Figure 1C and Table 1) [9]. Different sample sources at various geographic locations have their own bacterial community that can be targeted with different cultivation strategies. Thus, all the parameters resulted in a selected isolation. However, we did not observe a clear pattern or bias for the dominant phyla caused by any of the parameters (Supplementary Figure S2). Overall, different numbers of strains per phylum were obtained, which reflects the peculiarity of the untargeted high-throughput cultivation method. This untargeted isolation was used as an input for metabolomics, which is in contrast to the most common approach of selecting several strains that all correspond to a single phylogenetic group [28,29]. While the latter, common approach allows to identify and distinguish a core metabolome from special, often secondary metabolites in a narrow group, our study addresses the question of how far different phylogenetic groups share a consensus metabolome, or whether the phylogenetic distance is reflected by unique metabolites. For this purpose, the strains were fermented at a small, 100 mL scale to create biomass and provide organic extracts.

### 2.2. Untargeted Metabolomics Analysis

**Figure 2:**
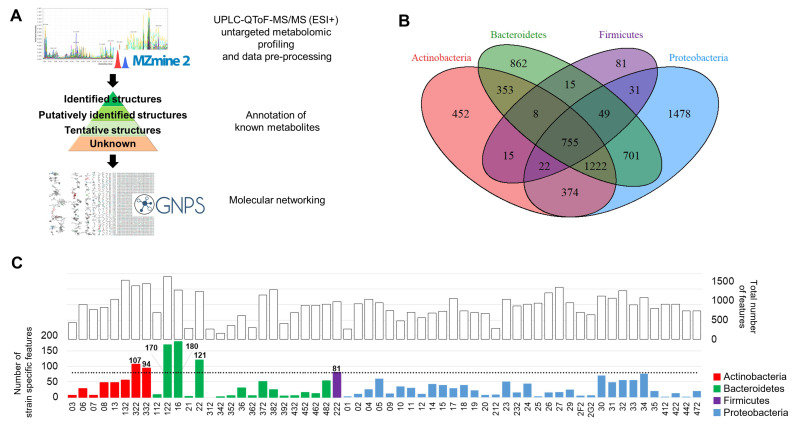
Global metabolome analysis across 60 bacterial strains. (**A**): Metabolomics experimental and data analysis workflow. (**B**): Venn diagram indicating the number of metabolomic features detected per phylum (total number of features: 6418). (**C**): Upper bar plot (white bars) depicting the total number of features detected per extract; lower bar plot depicting the number of strain-specific features that were only detected in the indicated extract (and in none of the other 59 extracts). The colour code indicates the bacterial phylum, and the dotted line depicts an arbitrarily chosen threshold of 80.

Endo-metabolites were extracted with acetone from 100 mL fermented cultures of the 60 difficult-to-grow bacterial samples. After drying and reconstituting the extracts, untargeted metabolomics using LC-MS/MS in the positive ion mode were performed in order to characterize the endo-metabolome chemical space. The collected data were processed and analysed with a workflow comprising the following steps (Figure 2A): (i) pre-processing of raw data with MZmine2 to list all the so-called molecular features defined by a unique combination of *m*/*z* and retention time (r.t.); (ii) annotation of known metabolites. This was performed consecutively by searching them against analytical standards present in our in-house library, followed by matching the MS/MS spectra with online databases, and finally through the structure prediction tool SIRIUS4 [30] coupled with CSI:Finger ID [31] (iii) generation of a molecular network through GNPS and chemical classification via ClassyFire, MolNetEnhancer, available from GNPS, and CANOPUS, available from SIRIUS (both tools are based on ClassyFire taxonomy).

A total of 6418 features were obtained from all 60 extracts after step (i) of the reported workflow, including isotopes, different adducts of the same metabolite and impurities from the extraction processes. For a first overview on the distribution of chemical diversity across phyla, the number of features detected in each phylum was plotted in a Venn diagram (Figure 2B) that visualized both features specific to each phylum as well as the number of features that were shared by two or more phyla. Of notice, only 11.7% of the features detected in the whole experiment (i.e., 755) were shared between all phyla (by at least one member per phylum). Of these, 340 features had a precursor ion mass of less than 300 Da, as is typical for primary metabolites. Almost half of the 6418 features (2873, i.e., 45%) were found to be specific to any one of the four phyla. Proteobacteria also displayed the highest specificity, with 1478 features detected only for this phylum, followed by Bacteroidetes (862 features), Actinobacteria (452 features) and finally by Firmicutes (81 features). This analysis indicates a large extent of unique metabolism of the investigated strains. The number of unique features per phylum strongly correlates (R = 0.99) with the number of isolates per phylum, indicating that the more different strains analysed, the more unique features and thus potential novel compounds can be detected. The investigated number of extracts was thus too small to reach saturation. On the single strain level, a large variability in the number of total features, and especially in the number of strain-specific features was observed (Figure 2C), identifying the most promising strains in terms of metabolite uniqueness. Notably, several strains within the Bacteroidetes displayed a high number of strain-specific features. In extracts 16, 122 and 22, more than 80 features specific to each of the strains were detected. In contrast, within the Proteobacteria phylum, no strain, out of the 35, had a high number of specific features. From the only strain belonging to Firmicutes, i.e., extract 222, 81 features were found to be strain-specific, with an overall number of 976 detected features. Among the eight extracts analysed from Actinobacteria, extracts 322 and 332 presented a high number of strain-specific features, whereas extract 132, which exhibited the second highest number of total features (1534, see top bar plot in Figure 2C) detected in the whole experiment, had a relatively low number of strain-specific features (only 56). Along this line, the number of specific features was zero in extract 312 and particularly low for extracts 342, 412 and 442 (2 features each), extracts 01, 21 and 25 (3 features each), extract 392 (4 features), extracts 352, 362, 432 and 2F2 (5 features each) and extracts 03, 07 and 2G2 (6 features each); these strains would be deprioritized in an in-depth follow-up investigation from a chemodiversity point of view.

Remarkably, among the extracts with more than 80 strain-specific features, extracts 322 and 332 from the Actinobacteria phylum displayed a very low percentage of similarity [27,32] to their closest relative strain (Table 1). In particular, with a similarity of 96.46% for AEG42_45 (extract 322) and of 96.86% for AEG42_13 (extract 332) to their next relatives “*Sporichthya brevicatena* AB006164” and “*Nocardioides humi* EF623863”, respectively, these two strains represent novel species in their respective genera and potentially even new genera. Thus, extracts 322 and 332 combine genetic distance with high metabolite specificity, and might therefore have a high potential for novel NPs. However, a clear correlation between taxonomic similarity to the next relative and presence of strain-specific features was not observed across the overall data set (Supplementary Figure S3). There is a trend that strains with a high similarity to their closest relative display a rather low specificity in terms of the number of detected features (yellow area in Supplementary Figure S3). However, exceptions to this trend exist, i.e., extracts 16, 122 and 22.

The highest number of strain-specific features in the whole set were detected for extract 16, corresponding to strain 3RW5_S4aa, and for extract 122, corresponding to strain M66, which produced, respectively, 180 and 170 features that were not found in any other extract (Figure 2C and Supplementary Figure S3). These two bacteria were identified as *Maribacter* (closest relative to strain 3RW5_S4aa is *M. litoralis MG456900* with 99.93% similarity) and *Flavobacterium* (closest relative to strain M66 is *F. terriphilum CUG00004 KT592306* with 99.12% similarity). A manifestation of the metabolome uniqueness, as observed within our analytical pipeline, would require a comparison with larger panels of closely related strains using the same methodology; this was beyond the scope of this study. Because multiple cultivation media were used, a subtraction of a standard medium blank was not possible. Thus, it is principally possible that some of the features are media components. However, we focused on the intracellular metabolomes, which are less impacted by the cultivation media than the exometabolome. Moreover, the large differences between samples cultivated with the same media demonstrates that the metabolome signatures are not dominated by media components.

We concluded that a highly diverse set of bacterial strains can be readily classified according to the overall number and the fraction of specific metabolic features they produce, with vast differences between and within phyla. However, within this diverse set, a correlation of taxonomic distance and uniqueness of metabolite features was not detectable.

### 2.3. Metabolite Annotation

**Figure 3:**
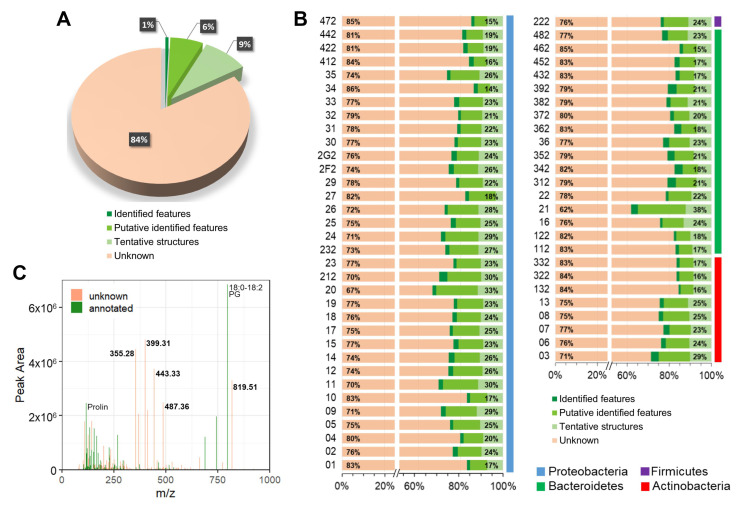
Metabolite annotation. (**A**): Pie chart of the whole experiment representing the percentage of (i) identified features (dark green), i.e., match of *m*/*z* and retention time vs. standards present in the in-house library; (ii) putatively identified ones (green), i.e., match of MS/MS spectrum vs. online databases, such as GNPS; (iii) tentatively assigned ones (light green), i.e., based on in silico evaluation of isotope pattern and fragmentation tree from SIRIUS4; (iv) unknowns (orange). (**B**): Bar plot of the percentage of known (green) and unknown (orange) features per each sample. Coloured bars on the right represent the four different phyla. Data labels on the right-hand side indicate the sum of percentages of all green features, whereas the label on the left-hand side indicates the percentage of unknown features. (**C**): Abundances of identified (in green) and unidentified (in orange) features in extract 21 were plotted in an *m*/*z* scan, with the peak area on the y-axis; labels of *m*/*z* values or compound annotation, where dereplicated, are displayed for the most abundant peaks. Extract 21 was the extract with the highest percentage of identified features.

Dereplication, or metabolite annotation, is a prerequisite for uncovering meaningful biological information from the acquired data. To distinguish known metabolites from potentially new ones in the investigated set, a dereplication of the 6418 features following a systematic protocol was conducted. This work resulted in the annotation of 1052 (16.4% of all) features (Figure 3A and Supplementary Data Table S4), with different confidence levels of metabolite identification [33,34].

In particular, 0.6% of the features were identified structures (confidence level 1), i.e., their identity was confirmed from a match of precursor ion, MS/MS spectrum and retention time, from pure reference standards present in the in-house library and acquired under identical analytical conditions. An additional 6.6% were putatively identified features (confidence level 2), i.e., exhibiting accurate precursor masses and MS/MS fragments consistent with externally acquired spectra present in online databases, such as GNPS and MassBank of North America. Finally, 9.2% of the detected features produced tentative structures (confidence level 3) when their accurate masses, isotopic distribution patterns and fragmentation trees were calculated from in silico structure prediction software (SIRIUS4 coupled with CSI:FingerID. Thus, the in silico evaluation allowed to significantly increase the annotation of known features; even if only tentative structures were proposed, completely unknown and potentially known molecules could be discriminated.

To evaluate the annotation accuracy of our approach, we compared the results obtained with the present workflow to those obtained by CluMSID, an MS/MS similarity-based method previously developed in our group [35] followed by a manual assignment, using a dataset obtained from a *P. aeruginosa* PA14 cell extract (see the Supplementary Material). While the two methods commonly identified 80 metabolites, 24 additional metabolites were only found by the untargeted method and 45 only by CluMSID/manual interpretation. A manual re-analysis of the additional 24 metabolites confirmed that all were annotated in a correct manner, while a majority of the missed 45 were detected in a knowledge-based, semi-targeted manner. This demonstrates that the annotation workflow described in this work generates metabolite assignments of a quality that is comparable to that of a hand-curated data analysis.

Of the annotated features in the present data set, 199 were found ubiquitously produced in all four phyla (by at least one strain per phylum) while 321 features were found exclusively in any one of the four phyla. Considering the dereplication depth at a strain-level, 18 strains had 25% or more of annotated features (Figure 3B). Extract 21 was the sample with the highest ratio of annotated features (37.8%), with 108 dereplicated features from 286 detected. We hypothesized that known features were of rather high abundance (and have therefore been noticed and identified before), whereas unknown features were of low abundance (and have been therefore overseen so far). To probe this, the peak area of each feature in extract 21 was plotted versus its mass-to-charge ratio, thereby reducing the LC/MS run to a single mass spectrum (Figure 3C). From this plot, it was evident that there was no correlation between feature abundance and annotation capability. Thus, many among the most abundant features could not be assigned to a metabolite.

In 20 extracts among the whole collection, more than 80% of all detected features remained unidentified, irrespective of the extensive effort to expand the annotation. This result suggests the high potential of these bacteria strains for the production of novel molecules. Among these 20 extracts, 132 and 362 were the only ones displaying a high rate of unknowns (84.5% and 82.7%, respectively) and also representing new genera in their respective families (Table 1). However, overall, a correlation between the share of non-annotated features, thus potentially new molecules, with phylogenetic novelty could not be observed.

### 2.4. Metabolite Distribution and Chemical Richness

Next, we examined the chemical nature of the metabolites that were dereplicated in the whole data set. For this purpose, they were classified according to chemical taxonomy rules with ClassyFire [36] an open access tool that covers 4820 classes of organic and inorganic compounds (http://classyfire.wishartlab.com/, accessed on 18 January 2021). Based on this analysis, the highest number of known molecules present in our collection of strains belonged to the class of “carboxylic acids and derivatives”, including the subclass of “amino acids, peptides, and analogues” (Supplementary Figure S4). This is not surprising, as amino acids are vital molecules in all kingdoms of life, providing the building blocks of proteins. Other well represented classes in the data set were fatty acyls. Bacteria cell membranes are the primary source of lipids; it is known that bacteria can control the biophysical characteristics of their phospholipidic membrane by adjusting its composition with different types of fatty acids that are produced from the alteration of the structures of pre-existing phospholipids; this behaviour allows them to survive and adapt to changes in environmental conditions, particularly temperature [37,38,39]. The fatty acyl characteristics of lipids rather than the headgroup, can promote membrane fluidity, for example when branching, double bonds, or cyclopropyl modifications are present, or rigidity, when saturated straight-chain fatty acids are present [40].

Interestingly, 4-methoxychalcone, belonging to the chemical class of “linear 1,3-diarylpropanoids”, was detected only in three strains, i.e., two Bacteroidetes, both belonging to the genus *Flavobacterium* (extracts 112 and 122) and in one Actinobacterium of the genus *Rubrobacter* (extract 132). 4-Methoxychalcone is a chalcone derivative that has shown diverse pharmacological properties, including anti-tumour and anti-inflammatory activities [41,42]. Ecologically, it may play a role in the chemical communication during biofilm (de-)formation. More precisely, 4-methoxychalcone has been reported to show antimicrobial activity by damaging the bacterial cell membrane and inhibiting slime-producing microorganisms [43,44]. Because 4-methoxychalcone is a known plant metabolite isolated from *Ficus lyrata*, its assignment from bacterial sources is remarkable. However, the synthesis of structurally identical aromatic polyketides from plants and bacteria, including chalcones such as naringenin, has been reported before [45,46]. Therefore, the microbial biosynthesis of 4-methoxychalcone is principally conceivable. 

The analysis of chemical richness and chemical nature presented above was based exclusively on the 1052 dereplicated features of the study. However, even after extensive dereplication, there was still a large fraction of features (84%) that were not annotated.

**Figure 4:**
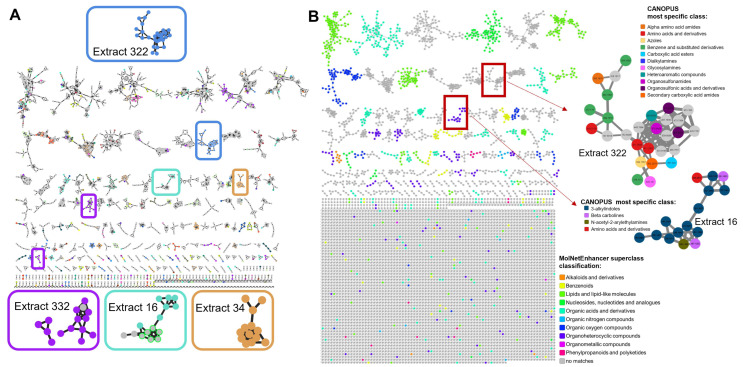
Feature-based molecular network and chemical classification. (**A**): FBMN (singletons are omitted in the figure) obtained from GNPS and visualized in Cytoscape. Strain-specific nodes (i.e., nodes detected in only one strain) are coloured according to their provenance strain (for colour legend, see Supplementary Figure S5). Subnetwork families where (almost) all unknown nodes derive exclusively from one extract are enlarged in the boxes. (**B**): FBMN coloured according to MolNetEnhancer chemical classification of superclass (based on ClassyFire). (**C**): (Top) Enlargement of subnetwork family of 28 nodes detected only in extract 322 and non-dereplicated. (Bottom) Enlargement of subnetwork family of 15 nodes detected only in extract 16, indicating indole-containing metabolites. Nodes are coloured according to the most specific classes obtained from CANPOPUS analysis.

To have a broader overview on the chemical space detected in the 60 strains, and illuminate the major chemical classes present, a feature-based molecular network (FBMN) was created in GNPS [18,47] and visualized with the software Cytoscape [48] (Figure 4A). The FBMN gives an overview on feature similarities that were detected in the whole experiment, regardless of a metabolite annotation. Each feature is represented by a node, characterized by its mass-to-charge ratio, its retention time, and its corresponding MS/MS fragmentation spectrum. Nodes were connected by edges if their MS/MS spectra were similar to each other, i.e., when they shared at least four common fragment ions and had a cosine score of 0.5 or more. This reflects a presumed chemical relatedness of the connected nodes.

The features clustered based on MS/MS spectral similarity were further analysed with the MolNetEnhancer program that propagates chemical class annotations to the full subnetwork in a semiautomated manner [49]. This approach requires an annotated node within the subnetwork family. Therefore, the information from the MolNetEnhancer was complemented by CANOPUS, a computational tool recently developed and integrated into the SIRIUS4 pipeline, which uses a deep neural network to predict compound classes from fragmentation spectra, and targets in particular features where spectral and structural references are not available [50].

The 6418 features detected in this work were organized into 292 subnetwork families comprising at least two nodes; the remaining 4456 (69%) features were singletons, meaning that they had a distinct MS/MS spectrum that was not clustered with any other one from the data set.

An ion identity molecular network (IIMN) [51] was also generated to reduce the complexity and redundancy of the FBMN by combining unconnected singletons and by collapsing multiple ions of the same molecule into single representative nodes. The collapsed network indeed presented a lower number of nodes (5054), but IIMN was not successful in reducing the number of singletons in the network, with a 65.3% share of unconnected nodes. This is possibly due to the raw data acquisition parameters, where the acquisition of MS/MS scans was favoured over survey scan frequencies, resulting in lower possible correlation of MS1 and thus connectivity in the molecular network. The high collision energy setting, which was selected to increase the number of MS/MS fragments generated for a given precursor ion, might also have contributed to the resulting high number of self-looped nodes [52].

Interestingly, the FBMN visualization points to the presence of metabolites produced and detected in only certain strains, as exemplified by a subnetwork found in extract 322 (Figure 4A): all 28 nodes from the subnetwork (depicted in blue) were exclusively detected in this sample. None of these nodes was dereplicated, indicating that a full family of potentially novel metabolites was present in this strain. The similarities are not easily recognized by eye, because the masses of the nodes are distributed in a range between 152–575 Da, and their retention times span between 1.4 and 10.5 min, with the majority being above 6 min. Finally, the peak areas cover a >30 fold range from 2 × 10^4^ to 7 × 10^5^. Nevertheless, the unique molecular features for extract 322 should be confirmed with repeat analysis of new extracts of the same strain, which could not be conducted in the current investigation due to the very limited biomass available.

While no matches could be assigned for the 28 nodes of the subnetwork family described above from the MolNetEnhancer workflow, CANOPUS predicted the presence of mainly carboxylic acids and derivatives (among which there were especially amino acids and derivatives, a secondary carboxylic acid amide and a carboxylic acid ester feature). Other well-represented subclasses were organosulfonic acids and derivatives and benzene and substituted derivatives (Figure 4C). Extract 322 has already emerged from the previous analysis as one of the samples with the highest number of specific features within the whole data set (Figure 2C). Moreover, with 84% of unknown features, it was also among the samples with a high potential for chemical novelty. The FBMN confirmed this assessment. Collectively these findings indicate that extract 322 is a priority candidate for an in-depth isolation effort.

A similar situation was found in extracts 332 (purple nodes in Figure 4A), 16 (light blue nodes in Figure 4A) and 34 (orange nodes in Figure 4A): the FBMN highlighted small clusters of non-de-replicated nodes detected almost exclusively from one of these three strains, which were already noted for their high number of strain-specific features (exacts 332 and 16) and ≥80% of unknown features (extracts 332 and 34).

Apart from focusing on potentially novel structures, a different goal may be to search for metabolites from specific chemical classes. In extract 16, several nodes were part of a larger cluster with representatives of indole derivatives (Figure 4C). Moreover, CANOPUS analysis identified these nodes as 3-alkylindoles or beta carbolines; thus, an isolation effort from a scale-up cultivation of this sample is predicted to yield organoheterocyclic compounds, and in particular indole-containing metabolites.

## 3. Conclusions

This study reports a workflow and several data analysis tools that can be applied to systematically explore the metabolomes of 60 newly isolated difficult-to-cultivate marine and soil bacteria from various sources and geographical locations. Experimental LC-MS/MS data were analysed with open-access tools in order to guide future targeted isolation efforts on selected strains and to improve the chances of finding potentially novel NPs. The de-replication workflow allowed to identify 1052 known molecules; however, the vast majority (84%) of metabolic features could not be assigned. This finding reflects an overlay of two phenomena: there are methodological limitations in data acquisition and analysis that lead to assignment failures, i.e., previously reported molecules are not recognized based on their (often non-trivial) LC-MS/MS signals [53]. Moreover, the retrieval of published mass spectral data from journal archives is often time consuming or impossible since they are reported only as figures. In addition, the large portion of unassigned features reflects the probably novel chemical compounds produced by such organisms. This is in line with the observation that the primary and secondary metabolomes of different species differ substantially [54,55]. We demonstrated that many features occur in only a single phylogroup or are even unique for a single strain. A part of the unidentified features was highly abundant, which suggests that the isolation of the corresponding compounds might be technically feasible. Technically, it is noteworthy that dereplication using a standard matching to internal and public databases was successful in only 7% of cases. The study illustrates that prediction tools like SIRIUS4 or molecular networks reflecting spectral similarity gave a substantial and required improvement in metabolome description. The further development of such tools, actively pursued currently [15], is clearly warranted.

Due to the high diversity of the microbial strain collection and its limited size, saturation effects were not yet visible, and likely novel metabolic features were observed from taxonomically new as well as known species at similar rates; this means, on the other hand, that genomic distance alone was not a sufficient pre-selection criterion. However, individual strains differed vastly by the overall number and the fraction of potentially novel metabolic features they produce, illustrating the importance of a pre-selection before large-scale cultivation and isolation.

In addition, the prediction tools yield a chemical structure classification, and they pinpoint to clusters of related metabolites within a strain. Compared to classical natural product dereplication procedures, the methods are unbiased, more comprehensive, and substantially faster. While this is achieved without any need of prior genomic knowledge, we anticipate that coupling metabolome and genomic information on biosynthetic gene clusters should yield an even better and more informed prediction [56].

Overall, we demonstrated that the metabolomics cascade established here—from untargeted data analysis via database matching to prediction and clustering—is a powerful method to classify microbial strains from large collections and prioritize samples for isolation, thereby fuelling the discovery of novel natural products.

## 4. Materials and Methods

### 4.1. Samples Collection

Marine samples of sea water, sediment and sponges from the Atlantic Ocean, Mediterranean Sea, Baltic Sea, Channel Sea and Pacific Ocean were collected during different sampling campaigns between 2014 and 2018. Seawater samples were collected close to the water surface (1 m depth) and about 10 m depth (Channel Sea and Baltic Sea) in sterile Nalgene bottles. Marine sediments were sampled in sterile 50 mL reaction tubes by divers or via the use of a small crane with a sediment grabber and directly transferred to a sterile 50 mL reaction tube. Samples were kept at 4 °C and processed within 10 h after sampling. Subsamples were fixed in 2% (*v*/*v*) glutaraldehyde for subsequent cell counting. For bacterial isolation, sediment samples were dispersed by vortexing in 10 mM 4-(2-hydroxyethyl)-1-piperazineethanesulfonic acid (HEPES) buffered at pH 7.3. Soil samples were collected in Brandenburg, Thüringen and Baden-Würtemberg in the framework of the Biodiversity Exploratories field campaign in May 2014 [57]. Isolates from marine hosts were obtained by direct plating (algae and sponge) and chemotaxis experiments (sponge). Isolate Rhodobacteraceae bacterium D100-Iso2 was isolated from a cyanobacterial culture from the Saltern ponds of Trapani, Sicily (in the framework of the EMBRIC project). Rhodobacteraceae sp. MEBiC05055, was isolated from a marine sponge in Geomun-Island, Korea. The respective algae (given in parenthesis) were provided by the Culture Collection of Algae at Goettingen University (SAG): *Sulfitobacter porphyrae* A11D-105 (isolated from /*Prorocentrum micans*/ SAG 2018, Dinophyta), *Sulfitobacter pseudonitzschiae* C05C-116 (isolated from /*Pyrenomonas salina*/ SAG 2002, Cryptophyta), *Hoeflea* sp. C05C-110 (isolated from /*Pyrenomonas salina*/ SAG 2002, Cryptophyta). The following strains were taken from the DSMZ: *Sulfitobacter dubius* DSM 16472^T^ (isolated from /*Zostera marina*/, sea grass), *Marinovum algicola* FF3 DSM 10251^T^ (isolated from /*Prorocentrum lima*/, Dinophyta), *Marinovum algicola* DG898 DSM 27768 (isolated from /*Gymnodinium catenatum*/, Dinophyta).

### 4.2. Cultivation Strategies

For bacterial isolation, four complementary strategies were applied in order to maximise the diversity of isolated strains (Figure S1).

#### 4.2.1. Single Dilution High-Throughput Cultivation in Liquid Media

This strategy applied a high-throughput cultivation approach based on (i) liquid oligotrophic media, (ii) a low concentration of inoculum in order to outcompete less abundant but fast-growing bacteria and (iii) long incubation times. These three factors allowed accessing difficult-to-grow bacteria [58,59,60].

Parallel liquid cultures were set up in 96-well microtiter plates (Figure S1). Before inoculation, total bacterial cell numbers were determined for each natural sample by fluorescence microscopy after staining with SYBR Green I (Life Technologies, Ltd., Paisley, UK). Each well of the microtiter plates was filled with 180 µL of medium and subsequently inoculated with 20 µL of inoculum containing 10 or 50 cells [61]. The plates were filled and inoculated either by hand using multichannel pipettes or automatically using the Thermo Scientific™ Multidrop™ Combi Reagent Dispenser (Waltham, MA, USA). The outer wells of each plate (36 wells) were not inoculated and served as negative controls. Five different culture media were used for the bacterial enrichment and isolation (Table S1): (i) DSMZ medium 1649 Artificial Sea Water (ASW) salts—including yeast and glucose (HD; 1:10 diluted); (ii) DSMZ medium 1649 Artificial Sea Water (ASW) salts -HD (1:10 diluted) Polymer; (iii) medium “insoluble humic analogs” (iv) medium “soluble humic analogs” and (v) DSMZ medium 1426 Soil Solution Equivalent (SSE)/HD 1:10, (Additional Information). Plates were incubated at 15 °C in the dark for 6–12 weeks. After incubation, the bacterial community grown in each well was analysed by a barcoded Illumina paired-end sequencing method targeting the 16S ribosomal RNA V1-2 hypervariable region [62]. The taxonomy of the reads was assigned against the SILVA database (v.128) [63] with UCLUST [64]. According to the taxonomic structure of the bacterial community of each well, a selected isolation strategy was carried out. Aliquots of each culture were plated on the above described medium solidified with 0.8% gelrite (*w*/*v*) (SERVA, Heidelberg, Germany). After incubation for 4–6 weeks, several representative colonies were picked from each plate and purified by three additional passages on the corresponding solidified medium.

#### 4.2.2. Direct Plating Method

The direct plating method was used for the enrichment of slow-growing bacteria which required a solid surface for growth. This approach is limited to bacteria able to produce (micro-)colonies on solid media. Five different culture media solidified with 0.8% gelrite (*w*/*v*) or 1.5% agar (*w*/*v*) were used for the bacterial enrichment (Table S1): (i) DSMZ medium 1649 Artificial Sea Water (ASW) salts -HD (1:10 diluted); (ii) DSMZ medium 514 Medium BACTO MARINE BROTH; (iii) L1ZM10; (iv) medium Soil Solution Equivalent SSE/HP and (v) DSMZ medium 1426 Soil Solution Equivalent (SSE)/HD 1:10 (Additional Information). Experiments were carried out in 90 mm Ø Petri dishes (Figure S1). Tenfold serial dilutions of the natural samples were performed in the corresponding medium. Subsequently, 100 µL of the 10^−3^ to 10^−6^ dilution was added to the culture medium surface and spread with a Drigalsky spatula. Plates were incubated at 15 °C in the dark for 6–12 weeks. After incubation, several representative colonies were picked from each plate and purified by three additional passages on the corresponding solidified medium.

#### 4.2.3. Growth in Biofilms

For the enrichment and isolation of biofilm-forming bacteria, the methodology described by Gich et al. [65] was used and adapted to marine samples. Solid, inert surfaces may lead to the stimulation of cell division and growth of starved bacteria [10,66]. Strips consisting of different, largely inert, solid materials (stainless steel, glass, polypropylene and polystyrene) were employed and incubated in 20 mL glass vials (Figure S1). Solid surfaces were incubated in 3 different media (Table S1): (i) DSMZ medium 1649 Artificial Sea Water (ASW) salts -HD (1:10 diluted); (ii) KM14 and (iii) DSMZ medium 514 Medium BACTO MARINE BROTH, and inoculated with 1000 cells from the natural samples. Vials were incubated at 15 °C for 8 weeks. To exert a selection pressure towards biofilm-forming microorganisms, three sequential enrichments were done. A sample was incubated with one strip, the colonized first strip was transferred to a second vial containing a sterile second strip and after its colonization, the second strip was transferred to yet another vial containing a sterile third strip. The solid surface strips were transferred to fresh medium every third month and the cultures incubated at room temperature. Finally, the biofilm that formed on the surface of the strips was spread onto the corresponding media and cultures were purified by subsequent re-streaking.

#### 4.2.4. Chemotaxis Chambers

Another approach for the selective enrichment and subsequent isolation of novel types of bacteria exploited the chemotactic responses of bacteria to specific substrates [67,68,69]. Although restricted to motile and chemotactically active microorganisms, a considerable fraction of species can be recovered with this technique, particularly in bacterioplankton communities. For the chemotaxis assays, glass capillaries loaded with defined substrate solutions were inserted in a suspension of motile microorganisms, and the accumulation of cells at the opening of or within the capillary was monitored by light microscopy. The substrates used for the isolation of marine bacteria are listed as Additional Information. Experiments were set up in small microscopic chambers (Figure S1; modified from Overmann, 2005 [69]), which were prepared using small 21 × 21 × 0.17 mm coverslips as spacers between the microscope slide and the lid, which consisted of another 60 × 24 × 0.17 mm coverslip. Spacers and the lid were fixed by sealing the two short and one long edges of the chamber with a paraffin/mineral oil mixture (4:1, *v*/*v*). Flat rectangular glass capillaries with a length of 50 mm, an inside diameter of 0.1 × 1.0 mm, and a capacity of 5 µL (Vitrocom, Mountain Lakes, NJ, USA) were used. These capillaries fit exactly into the opening of the chemotaxis chamber. The specific geometry of these capillaries permitted direct light microscopic examination of their contents. For marine samples, the small microscopic chambers were incubated at room temperature for 3 h. After incubation, the capillaries are removed from the chambers. For direct microscopy of the accumulated microorganisms, the open end of each capillary was immediately sealed with plasticine. Subsequently, bacterial cells trapped in the capillaries could be transferred to Petri dishes or 96-multiwell plates filled with DSMZ medium 1649 Artificial Sea Water (ASW) salts -HD (1:10 diluted) exerting positive pressure with a pipette from one end of the capillary.

### 4.3. Taxonomic Affiliation of Isolates

The taxonomic affiliation of all axenic bacterial isolates was investigated by sequencing their 16S rRNA gene. The almost full-length 16S gene of strains was amplified directly by colony-PCR using the primer pair 8f (5′-AGAGTTTGATCCTGGCTCAG-3′) [70] and 1492r (5′-GGTTACCTTGTTACGACTT-3′). PCR mixtures included 2.0 µL PCR buffer (10×), 0.8 µL MgCl2 (25 mM), BSA 0.4 µL (20 mg mL^−1^), 0.4 µL dNTPs (10 mM each), 0.08 µL each forward and reverse primers (50 pmol µL^−1^), 0.08 µL Dream Taq DNA polymerase (5 U µL^−1^ Thermo Scientific) and 1.0 µL template (picked colonies were added to 20 µL of water followed by three freeze/thaw cycles (−20 °C/microwave oven)) in a total volume of 20 µL. The thermal cycling program consisted of: (i) 10 min at 94 °C; (ii) 32 cycles of 30 s at 94 °C, 30 s at 56 °C and 1 min at 72 °C, and (iii) a final elongation step of 7 min at 72 °C. PCR products were purified and sequenced using the above primer pairs and the internal primers 1055f (5′-ATGGCTGTCGTCAGCT-3′) [71] and 341r (5′-CTGCTGCCTCCCGTAGG-3′) [72] and by Sanger sequencing employing the AB 3730 DNA DNA analyser (Applied Biosystems, Foster City, CA, USA) and the AmpliTaq^®^ FS BigDye^®^ Terminator Cycle Sequencing Kit (Applied Biosystems, Foster City, CA, USA). Subsequently, the 16S rRNA sequences were analysed with the online database EzBioCloud [73].

Pairwise sequence similarities were calculated using the method recommended by Meier-Kolthoff et al. [74]. Sequences were uploaded to the online webserver Genome-to-Genome Distance Calculator available at http://ggdc.dsmz.de/ accessed on 17 May 2022 [75] using the online submission form to determine single-gene trees (phylogeny server) [76] and the obtained 16S rRNA sequences of the 60 isolates as multi-FASTA file format as query and reference [77]. Phylogenies (trees and similarities) were inferred by the GGDC web server [75] available at http://ggdc.dsmz.de/ accessed on 17 May 2022 using the DSMZ phylogenomic pipeline [76] adapted to single genes. A multiple sequence alignment was created with MUSCLE [78]. maximum likelihood (ML) and maximum parsimony (MP) trees were inferred from the alignment with RAxML [79] and TNT [80], respectively. For ML, rapid bootstrapping in conjunction with the autoMRE bootstopping criterion [81] and subsequent search for the best tree was used; for MP, 1000 bootstrapping replicates were used in conjunction with tree-bisection-and-reconnection branch swapping and ten random sequence addition replicates. The sequences were checked for compositional bias using the Χ² test as implemented in PAUP* (* Phylogenetic Analysis Using PAUP) [82,83]. The input nucleotide matrix for the maximum likelihood phylogenetic tree comprised 60 operational taxonomic units and 1591 characters, 864 of which were variable and 762 of which were parsimony informative. The base-frequency check indicated no compositional bias (*p* = 0.74, α = 0.05). ML analysis under the GTR+CAT model yielded the highest log likelihood of −24,049.98, whereas the estimated alpha parameter was 0.34. The ML bootstrapping converged after 650 replicates; the average support was 79.49%, MP analysis yielded a best score of 5074 (consistency index 0.33, retention index 0.66) and 2 best trees. The MP bootstrapping average support was 80.65%. The tree was plotted using the Interactive Tree Of Life (iTOL) v6 [84].

### 4.4. Fermentation of Bacteria for Natural Product Analysis

For the fermentation of strains, 1 × 100 mL cultures containing liquid ASW/HD 1:10 (in 250 mL Erlenmeyer flasks) were inoculated with 3.0 mL (3% *v*/*v* final culture volume) of a seed culture. Depending on the growth kinetics and optimum condition of each strain, the cultures were fermented at 15–28 °C for 3–5 days, on a rotary shaker at 180 rpm.

After fermentation, the well-grown culture (1 × 100 mL) was sieved through a metal sieve (mesh size 270 µm). The biomass was added to Erlenmeyer flasks containing 70 mL of acetone and shaken at 180 rpm at 21 °C in a dark chamber for 3 h. The acetone was then filtrated through a folded filter into a 250 mL round-bottomed flask and dried in a rotavapor at 44 °C. Biomasses were reconstituted in acetonitrile at a concentration of 0.5 mg/mL.

All solvents used were Baker Analyzed™ Ultra LC/MS grade (Fisher Scientific, Schwerte, Germany).

### 4.5. Untargeted Metabolomic Profiling

All reconstituted extracts were analysed by ultra-high-performance liquid chromatography—tandem mass spectrometry (UPLC-ESI-QToF-MS/MS) on a Bruker maXis HD QToF mass spectrometer, equipped with an Apollo II electrospray source (Bruker, Bremen, Germany), operated in positive electrospray ionization mode. The mass spectrometer was coupled to an UltiMate 3000 RS (Thermo Scientific Dionex) UPLC system, equipped with a Kinetex C18 reversed phase column (1.7 µm, 150 × 2.1 mm from Phenomenex, Aschaffenburg, Germany), for chromatographic separation of metabolites.

Sample injection volume was 5 µL, with a system flow rate of 300 µL/min; the system was kept at 40 °C. A 30 min gradient elution with water (+0.1% *v*/*v* formic acid) as eluent A and acetonitrile (+0.1% *v*/*v* formic acid) as eluent B, was run as follows: 1% B for 0 min to 2 min, linear gradient from 1% B to 100% B from 2 to 20 min, 100% B held until 25 min and linear gradient from 100% B to 1% B from 25 to 30 min.

Raw data were acquired in full scan mode (50–1500 Da) in a data dependent MS/MS mode, performing collision-induced fragmentation of the five most abundant ions in each MS scan, using Bruker’s “Smart Exclusion” (2×) functionality to minimize multiple fragmentation of the same ion. The collision energy was ramped from 80% to 200% of the default auto-MS/MS collision energy (CID interpolated list: mass = 100, width = 4, charge state = 1, collision energy = 20; mass = 500, width = 5, charge state = 1, collision energy = 35; mass = 1000, width = 6, charge state = 1, collision energy = 55; mass = 100, width = 4, charge state = 2, collision energy = 17; mass = 500, width = 5, charge state = 2, collision energy = 30; mass = 1000, width = 6, charge state = 2, collision energy = 50).

### 4.6. Data Processing and Metabolomics Analysis

#### 4.6.1. Feature Detection

Raw LC-MS/MS data had lock mass calibration applied and were converted into mzXML format using Bruker DataAnalysis and Bruker Compass Xport software.

The data processing software MZmine2 (version 2.37.1-corr17.7) was used for detection of chromatographic peaks and filtering of detected features (retention time—*m*/*z* pairs). Processing parameters to obtain the feature table, generating and exporting the mgf and quantification table to be used in GNPS and SIRIUS4 and for ion identity molecular networking in MZmine2 are reported in Table S2.

#### 4.6.2. Metabolite Annotation

Features were searched first against our in-house library, built with analytical standards from the MSMLS—Mass Spectrometry Metabolite Library of Standards (IROA Technologies, Bolton, MA, USA) as well as a number of individually bought compounds from Sigma-Aldrich (Taufkirchen, Germany). Identification was confirmed by matching the precursor mass, retention time and MS/MS spectrum values to the available standards.

Then, putative annotation of known metabolites was expanded through GNPS (https://gnps.ucsd.edu/, accessed initially on 3 October 2020) spectral library matching of mass values and MS/MS spectra (library spectra were required to have at least a score of 0.6 and 3 matched peaks; annotations were then manually filtered based on quality of library entry, biological knowledge of sample set and eventually validated by matching with other online spectral databases, such as MassBank of North America (https://mona.fiehnlab.ucdavis.edu/, accessed on 20 November 2020). MS2LDA [84tha] workflow was used to guide or confirm some putative annotation, through the analysis of common patterns of mass fragments and indication of neutral losses.

Finally, SIRIUS4 (version 4.9.12), a software framework for the analysis of LC-MS/MS data of metabolites, integrated with CSI:FingerID (both developed at developed at the Chair of for Bioinformatics, Jena, Germany), was used to propose tentative structures for known features that were not identified in the previous annotation steps. Molecular formulas are deduced in SIRIUS4 by ranking isotope patterns from mass spectra of high resolution; while structures were proposed through a combination of fragmentation tree computation and machine learning in CSI:FingerID. The parameters used to process the present data set were the following: for molecular formula calculation, possible ionization: [M + H]^+^, [M + Na]^+^, [M + K]^+^, instrument: Q-TOF, ppm tolerance: 10 ppm, top molecular formula candidates: 10, filter: formulas from databases: Natural Products, KNApSAcK, SuperNatural, COCONUT, CHEBI, ZINC bio and MeSH. For the CSI:FingerID step, the possible adducts were set to: [M + H]^+^, [M-H_2_O + H]+, [M + Na]^+^, [M + K]^+^ and [M + NH_4_]^+^. A structure prediction was considered correct and thus kept in the present analysis only when its CSI:FingerID score was smaller than −150 and the corresponding best molecular formula candidate had a Zodiac score larger than 60%.

Chemical classification according to ClassyFire taxonomy was accessed thought the web-based application for automated structural classification of chemical entities (http://classyfire.wishartlab.com/, accessed on 18 January 2021) for annotations of level 1 (derived from the in-house library) and through GNPS MolNetEnhancer [49] and SIRIUS4 CANOPUS [50] for annotations of level 2 and 3, respectively, as class annotation through ClassyFire is integrated and available via these tools.

#### 4.6.3. Molecular Networking

mzXML files, together with MS/MS spectra files mgf and feature table csv files from MZmine2 were uploaded to the Global Natural Products Social Molecular Networking (GNPS, http://gnps.ucsd.edu, accessed initially on 3 October 2020) online tool, and a FBMN was generated through the online workflow available from the GNPS website.

Data was filtered by removing all MS/MS fragment ions within +/−17 Da of the precursor *m*/*z*. The precursor ion mass tolerance was set to 0.02 Da and a MS/MS fragment ion tolerance of 0.02 Da. A network was then created where edges were filtered to have a cosine score above 0.5 and more than four matched peaks. Edges between two nodes were kept in the network only if each of the nodes appeared in each other’s respective top ten most similar nodes.

Finally, the maximum size of a molecular family was set to 100, and the lowest scoring edges were removed from molecular families until the molecular family size was below this threshold. The visualization and analysis of the obtained FBMN was conducted through Cytoscape software [48] version 3.9.1.

## Figures and Tables

**Table 1:** Overview of the 60 bacterial isolates that were selected for metabolomics analysis.

Extract ID	Strain ID	Accession Number	Source	Origin	Cultivation Strategy	Isolation Medium	Phylum	Genus	Closest Relative	Similarity (%)
01	HEG41_91	OP776843	Soil	German soil	Direct plating	SSE 1:10 HD	Proteobacteria	*Bradyrhizobium*	*Bradyrhizobium uaiense* UFLA03 164 KC879705	97.02 *
02	4RS2_G4	OP776844	Sediment	Channel Sea	Biofilm	ASWsalts 1:10 HD	Proteobacteria	*Sulfitobacter*	*Sulfitobacter dubius* DQ915635	99.67
03	JAB_HD_127b	OP776845	Water	Baltic Sea	Multiwell plate	ABWsalts 1:10 HD	Actinobacteria	*Rhodococcus*	*Rhodococcus qingshengii* JCM 15477 DQ090961	100.00
04	PCS2D_E11	OP776846	Sediment	Atlantic Ocean	Multiwell plate	ASWsalts 1:10 HD Polymer	Proteobacteria	*Oceanisphaera*	*Oceanisphaera psychrotolerans* KF418814	99.89
05	JAB_HD_128b	OP776847	Water	Baltic Sea	Multiwell plate	ABWsalts 1:10 HD	Proteobacteria	*Devosia*	*Devosia psychrophila* GU441678	98.83
06	JAB_HD_2a	OP776848	Water	Baltic Sea	Multiwell plate	ABWsalts 1:10 HD	Actinobacteria	*Rhodococcus*	*Rhodococcus qingshengii* JCM 15477DQ090961	100.00
07	JAB_HD_137a	OP776849	Water	Baltic Sea	Multiwell plate	ABWsalts 1:10 HD	Actinobacteria	*Rhodococcus*	*Rhodococcus jostii* KF410370	99.24
08	JAB_HD_121a	OP776850	Water	Baltic Sea	Multiwell plate	ABWsalts 1:10 HD	Actinobacteria	*Microbacterium*	*Microbacterium marinum* EF204420	100.00
09	4RS2_G3b	OP776852	Sediment	Channel Sea	Biofilm	ASWsalts 1:10 HD Glass	Proteobacteria	*Aliidiomarina*	*Aliidiomarina soli* KX548074	97.10
10	4RW5_PS1	OP776853	Water	Channel Sea	Biofilm	ASWsalts 1:10 HD Polymer	Proteobacteria	*Alteromonas*	*Alteromonas macleodii* AB681740	99.35
11	CS1_PP3	OP776854	Sediment	Atlantic Ocean	Multiwell plate	ASWsalts 1:10 HD Polymer	Proteobacteria	*Pseudoalteromonas*	*Pseudoalteromonas shioyasakiensis* AB720724	99.65
12	4CH2_twe	OP776855	Sponge	host	Chemotaxis	ASWsalts 1:10 HD	Proteobacteria	*Vibrio*	*Vibrio kanaloae* CAIM 485 MT757984	99.85
13	JAB_HD_4a2	OP776856	Water	Baltic Sea	Multiwell plate	ABWsalts 1:10 HD	Actinobacteria	*Aeromicrobium*	*Aeromicrobium ginsengisoli* AB245394	99.47
14	4RS2_G3a	OP776857	Sediment	Channel Sea	Biofilm	ASWsalts 1:10 HD Glass	Proteobacteria	*Halomonas*	*Halomonas alkaliphila* AJ640133	99.93
15	4RW5_PS3	OP776858	Water	Channel Sea	Biofilm	ASWsalts 1:10 HD	Proteobacteria	*Pseudovibrio*	*Pseudovibrio ascidiaceicola* AB681198	98.51
16	3RW5_S4aa	OP776859	Water	Channel Sea	Biofilm	ASWsalts 1:10 HD Steel	Bacteroidetes	*Maribacter*	*Maribacter litoralis* MG456900	99.93
17	JAB_HD_102a2	OP776860	Water	Baltic Sea	Multiwell plate	ABWsalts 1:10 HD	Proteobacteria	*Pseudomonas*	*Pseudomonas pelagia* strain CL-AP6 EU888911	98.79
18	4d1_twe	OP776861	Sponge	host	Chemotaxis	ASWsalts 1:10 HD	Proteobacteria	*Pseudomonas*	*Pseudomonas knackmussii* B13 AJ272544	99.67
19	4RS2_G7	OP776862	Sediment	Channel Sea	Biofilm	ASWsalts 1:10 HD Glass	Proteobacteria	*Lutimaribacter*	*Lutimaribacter pacificus* DQ659449	97.04 *
20	JAB_HD_109a	OP776863	Water	Baltic Sea	Multiwell plate	ABWsalts 1:10 HD	Proteobacteria	*Pseudorhodobacter*	*Pseudorhodobacter ponti* KX771233	97.15 *
21	RW5_G2	OP776864	Water	Channel Sea	Biofilm	ASWsalts 1:10 HD Glass	Bacteroidetes	*Altibacter-Rhodococcus*	*Rhodococcus yunnanensis* AY602219	99.33
22	CS1PS2a	OP776865	Sediment	Atlantic Ocean	Biofilm	ASWsalts 1:10 HD Polymer	Proteobacteria	*Paracoccus*	*Paracoccus indicus* MG845150	99.77
23	D100_Iso2	OP776866	Alga	host	Direct plating	MB	Proteobacteria	*Aquicoccus*	*Aquicoccus porphyridii* MF113254	96.82 *
24	MEBiC05055	OP776870	Sponge	host	Direct plating	MB	Proteobacteria	*Tateyamaria*	*Tateyamaria armeniaca* LC464518	98.34
25	DSM_16472T	OP776867	Water	Sea of Japan	Direct plating	MB	Proteobacteria	*Sulfitobacter*	*Sulfitobacter dubius* DQ915635	100.00 *
26	DSM_10251T	OP776871	Alga	host	Direct plating	MB	Proteobacteria	*Marinovum*	*Marinovum algicola* DG898 DSM 27768	100.00 *
27	DSM_27768	OP776872	Alga	host	Direct plating	MB	Proteobacteria	*Marinovum*	*Marinovum algicola* FF3 DSM 10251T	100.00 *
29	C05C_116	OP776869	Alga	host	Direct plating	L1ZM10	Proteobacteria	*Sulfitobacter*	*Sulfitobacter pseudonitzschiae* KF006321	99.50
30	A11D_105	OP776868	Alga	host	Direct plating	MB	Proteobacteria	*Sulfitobacter*	*Sulfitobacter porphyrae* AB758574	99.85
31	A05D_005	OP776873	Alga	host	Direct plating	MB	Proteobacteria	*Aquicoccus*	*Aquicoccus porphyridii* MF113254	100.00
32	C05C_110	OP776875	Alga	host	Direct plating	MB	Proteobacteria	*Hoeflea*	*Hoeflea alexandrii* MT760263	99.69
33	H01Y_008A	OP776874	Alga	host	Direct plating	MB	Proteobacteria	*Fretibacter*	*Fretibacter rubidus* FJ394547	97.12 *
34	RW5_G4	OP776824	Water	Channel Sea	Biofilm	ASWsalts 1:10 HD Glass	Proteobacteria	*Amylibacter*	*Amylibacter cionae* KX790330	99.19
35	JAB_HD_121b	OP776851	Water	Baltic Sea	Multiwell plate	ABWsalts 1:10 HD	Proteobacteria	*Pseudorhodobacter*	*Pseudorhodobacter wandonensis* JN247434	99.18
36	JAB_HD_38	OP776826	Water	Baltic Sea	Multiwell plate	ASWsalts 1:10 HD	Bacteroidetes	*Algoriphagus*	*Algoriphagus aquaemixtae* KY661386	99.26
112	M64	OP776831	Water	Baltic Sea	Biofilm	KM14	Bacteroidetes	*Flavobacterium*	*Flavobacterium circumlabens* P5626 MH100898	98.80
122	M66	OP776832	Water	Baltic Sea	Biofilm	KM14	Bacteroidetes	*Flavobacterium*	*Flavobacterium terriphilum* CUG00004 KT592306	99.12
132	M20	OP776827	Water	Baltic Sea	Biofilm	KM14	Actinobacteria	*Rubrobacter*	*Rubrobacter radiotolerans* X87134	93.95 **
212	M55	OP776829	Water	Baltic Sea	Biofilm	MB	Proteobacteria	*Altererythrobacter*	*Altererythrobacter epoxidivorans* DQ304436	97.94 *
222	M62	OP776830	Water	Baltic Sea	Biofilm	MB	Firmicutes	*Bacillus*	*Bacillus mobilis* MCCC 1A05942 KJ812449	99.93
232	M09	OP776828	Water	Baltic Sea	Biofilm	MB	Proteobacteria	*Altererythrobacter*	*Altererythrobacter aquiaggeris* KX812543	98.73
312	SEG27_38	OP776841	Soil	German soil	Direct plating	SSE 1:10 HD	Bacteroidetes	*Chitinophaga*	*Chitinophaga flava* MH553387	93.57 **
322	AEG42_45	OP776842	Soil	German soil	Direct plating	SSE 1:10 HD	Actinobacteria	*Sporichthya*	*Sporichthya brevicatena* AB006164	96.46 *
332	AEG42_13	OP776840	Soil	German soil	Direct plating	SSE 1:10 HD	Actinobacteria	*Nocardioides*	*Nocardioides humi* EF623863	96.86 *
342	ACS3D_E6	OP776819	Sediment	Atlantic Ocean	Multiwell plate	SSE 1:10 HD	Bacteroidetes	*Ulvibacter*	*Ulvibacter antarcticus* AB681898	97.28 *
352	HEG41_64b	OP776836	Soil	German soil	Direct plating	SSE 1:10 HD	Bacteroidetes	*Niastella*	*Niastella populi* EU877262	96.17 *
362	SEG27_44	OP776837	Soil	German soil	Direct plating	SSE 1:10 HD	Bacteroidetes	*Pseudoflavitalea*	*Pseudoflavitalea rhizosphaerae* KU379667	94.04 **
372	AEG42_46	OP776839	Soil	German soil	Direct plating	SSE 1:10 HD	Bacteroidetes	*Flavitalea*	*Flavitalea flava* KX762320	99.80
382	SEG27_28	OP776838	Soil	German soil	Direct plating	SSE 1:10 HD	Bacteroidetes	*Niveitalea*	*Niveitalea solisilvae* KX268597	92.80 **
392	AEG42_23	OP776835	Soil	German soil	Direct plating	SSE 1:10 HD	Bacteroidetes	*Ferruginibacter*	*Ferruginibacter yonginensis* MT760289	93.85 **
412	PCS2D_E7	OP776816	Sediment	Atlantic Ocean	Multiwell plate	ASWsalts 1:10 HD Polymer	Proteobacteria	*Marinomonas*	*Marinomonas atlantica* LN909522	99.86
422	CS3_PS3b	OP776818	Sediment	Atlantic Ocean	Biofilm	ASWsalts 1:10 HD Polymer	Proteobacteria	*Amylibacter*	*Amylibacter lutimaris* MF113253	99.85
432	3RW5_PP6	OP776825	Water	Channel Sea	Biofilm	ASWsalts 1:10 HD Polymer	Bacteroidetes	*Ulvibacter*	*Ulvibacter antarcticus* AB681898	96.77 *
442	ACS3C_E5	OP776817	Sediment	Atlantic Ocean	Multiwell plate	ASWsalts 1:10 HD	Proteobacteria	*Pseudoalteromonas*	*Pseudoalteromonas shioyasakiensis* SE3 AB720724	99.65
452	2CW3_G4	OP776820	Water	Atlantic Ocean	Biofilm	ASWsalts 1:10 HD Polymer	Bacteroidetes	*Balneola*	*Balneola vulgaris* AY576749	94.85 **
462	M68	OP776833	Water	Baltic Sea	Biofilm	ABWsalts 1:10 HD	Bacteroidetes	*Arenibacter*	*Arenibacter algicola* FJ176555	99.91
472	RS2_PS_4	OP776823	Sediment	Channel Sea	Biofilm	ASWsalts 1:10 HD Polymer	Proteobacteria	*Pararhodobacter*	*Pararhodobacter oceanensis* KY009733	99.85
482	M72	OP776834	Water	Baltic Sea	Biofilm	ABWsalts 1:10 HD	Bacteroidetes	*Algoriphagus*	*Algoriphagus jejuensis* EF217418	98.79
2F2	ARW1_2F2	OP776821	Water	Channel Sea	Multiwell plate	ASWsalts 1:10 HD	Proteobacteria	*Arcobacter*	*Arcobacter lekithochrous* LT629298	98.16 *
2G2	ARW1_2G2	OP776822	Water	Channel Sea	Multiwell plate	ASWsalts 1:10 HD	Proteobacteria	*Arcobacter*	*Arcobacter lekithochrous* LT629298	98.17 *

ID: Identification; SSE: Soil solution equivalent; ASW: Artificial sea water; MB: Marine broth; L1ZM10; KM14; HD: Yeast (Hefe) and glucose (dextrane) addition (Table S1); * potential new species; ** potential new genus.

## Data Availability

Raw instrument files, peak list files, quantification table and mgf result file are publicly available at ftp://massive.ucsd.edu/MSV000088967/, accessed on 3 October 2020. The FBMN of this project is available at: https://gnps.ucsd.edu/ProteoSAFe/status.jsp?task=6b66d703fc754102b07d4ffe303a0f93, accessed on 3 October 2020. Further additional processing of this FBMN are available at the following links. MS2LDA job: https://gnps.ucsd.edu/ProteoSAFe/status.jsp?task=4fda277f2e4344909da298ea34e89b11, accessed on 3 October 2020. MolNetEnhancer job: https://gnps.ucsd.edu/ProteoSAFe/status.jsp?task=cf409aea607341e6a5b690dd02a57b4a, accessed on 3 October 2020. IIMN job: https://gnps.ucsd.edu/ProteoSAFe/status.jsp?task=6feaa11ef0a64b388f1634f607d96944, accessed on 3 October 2020.

## Supplementary Materials

The following supporting information can be downloaded at: https://www.mdpi.com/article/10.3390/md20110713/s1. Figures S1–S5, Tables S1–S3, and supplementary text are combined in a single file. Table S4 is available as an additional xlsx file.
