# Supplementary material for: A Metabolomics-Based Toolbox to Assess and Compare the Metabolic Potential of Unexplored, Difficult-to-Grow Bacteria
Source: Mar Drugs. 2022 Nov 14;20(11):713. doi: 10.3390/md20110713 (PMC9698959; doi:10.3390/md20110713)
Supplement: Supplementary file 1 [file marinedrugs-20-00713-s001.zip › Supplementary Material_f.pdf]

Supplementary Materials

A metabolomics-based toolbox to assess and compare the metabolic potential of unexplored, difficult-to-grow bacteria

Federica Fiorini, Felizitas Bajerski, Olga Jeske, Cendrella Lepleux, Jörg Overmann, Mark Brönstrup

Supplementary Figures

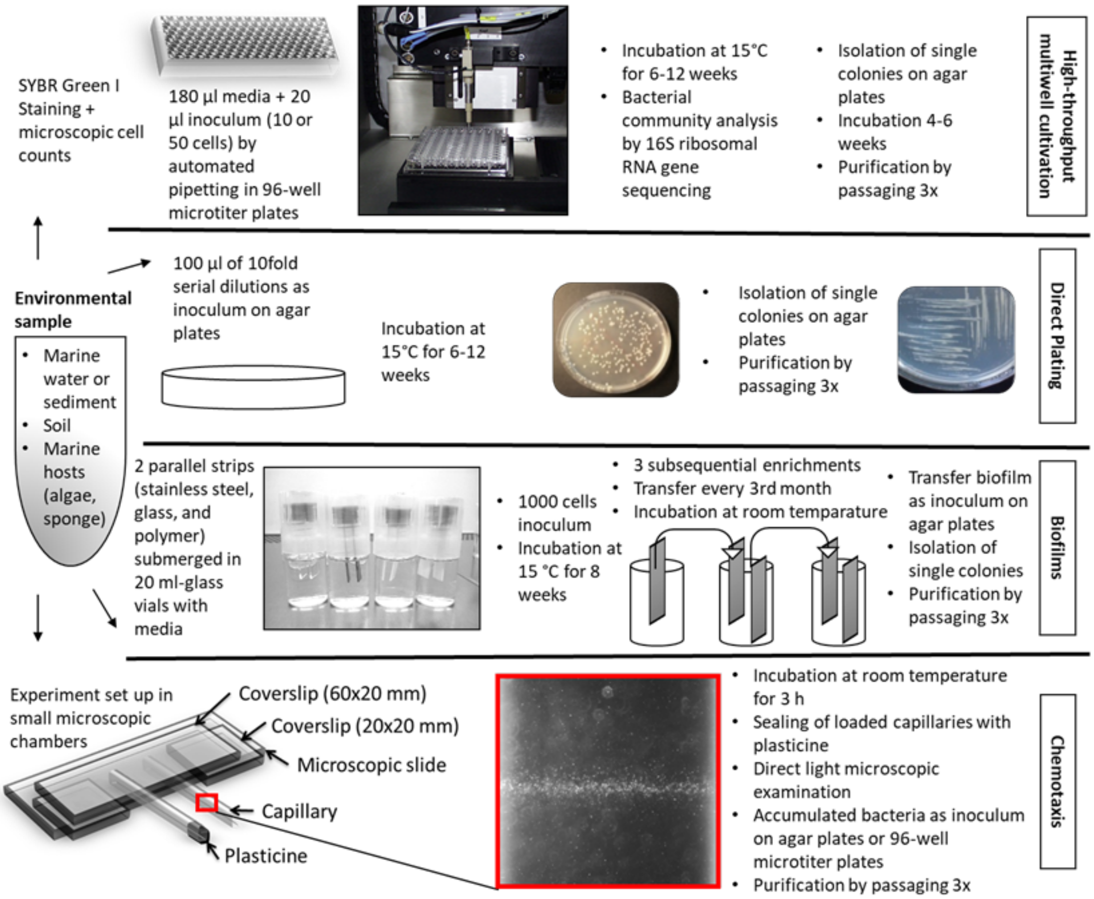

Figure S1. Cultivation strategies applied in this study to maximize the diversity of isolated strains**.** Marine samples (water, sediment, algae, sponges) from diverse regional origins and terrestrial samples from German soils were cultivated by four methods that comprised biofilm, chemotaxis, direct plating and high-throughput multiwell plate cultivation (see Table 1).

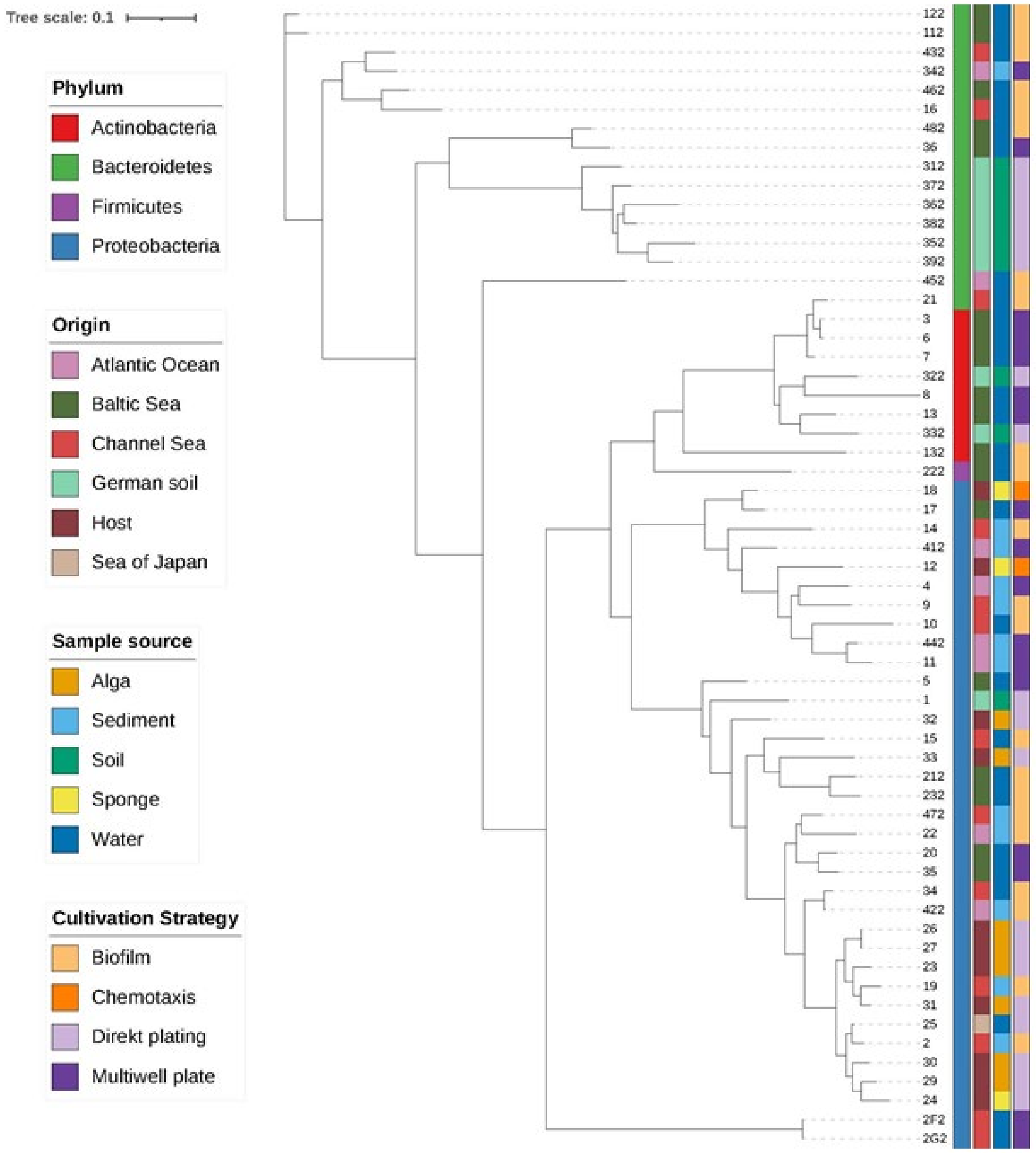

Figure S2. Maximum likelihood phylogenetic tree of 60 recalcitrant bacterial strains. The five columns on the right of the tree display sample numbers with respective phylum, origin, sample source and cultivation strategy (from left to right). The heterogeneous distribution pattern indicates that
every sampling site has its own bacterial community without causing a clear cultivation bias.

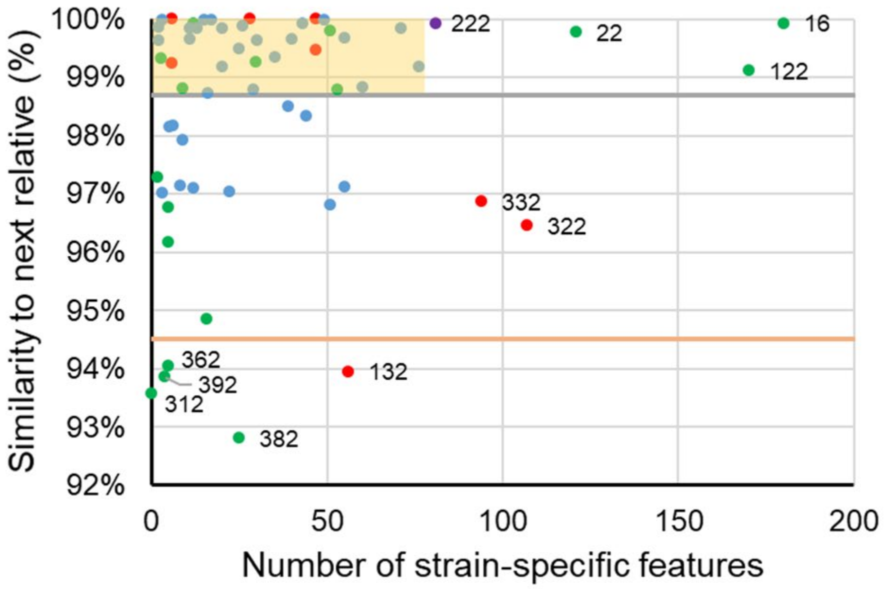

Figure S3. Taxonomic similarity to the closest relative in % vs. the number of strain-specific features. The grey line indicates the value of 98.7% 16S rRNA gene sequence similarity, that was used as threshold for differentiating two species, while the orange line is set at 94.5%, which differentiates two genera. Samples 332 and 322, which are among the extracts with more than 80 strain-specific features, display percentage of similarity to their closest relative strain lower than 98.7%. The color code indicates the bacterial phylum (blue for *Proteobacteria*, purple for *Firmicutes*, green for *Bacteroidetes* and red for *Actinobacteria*).

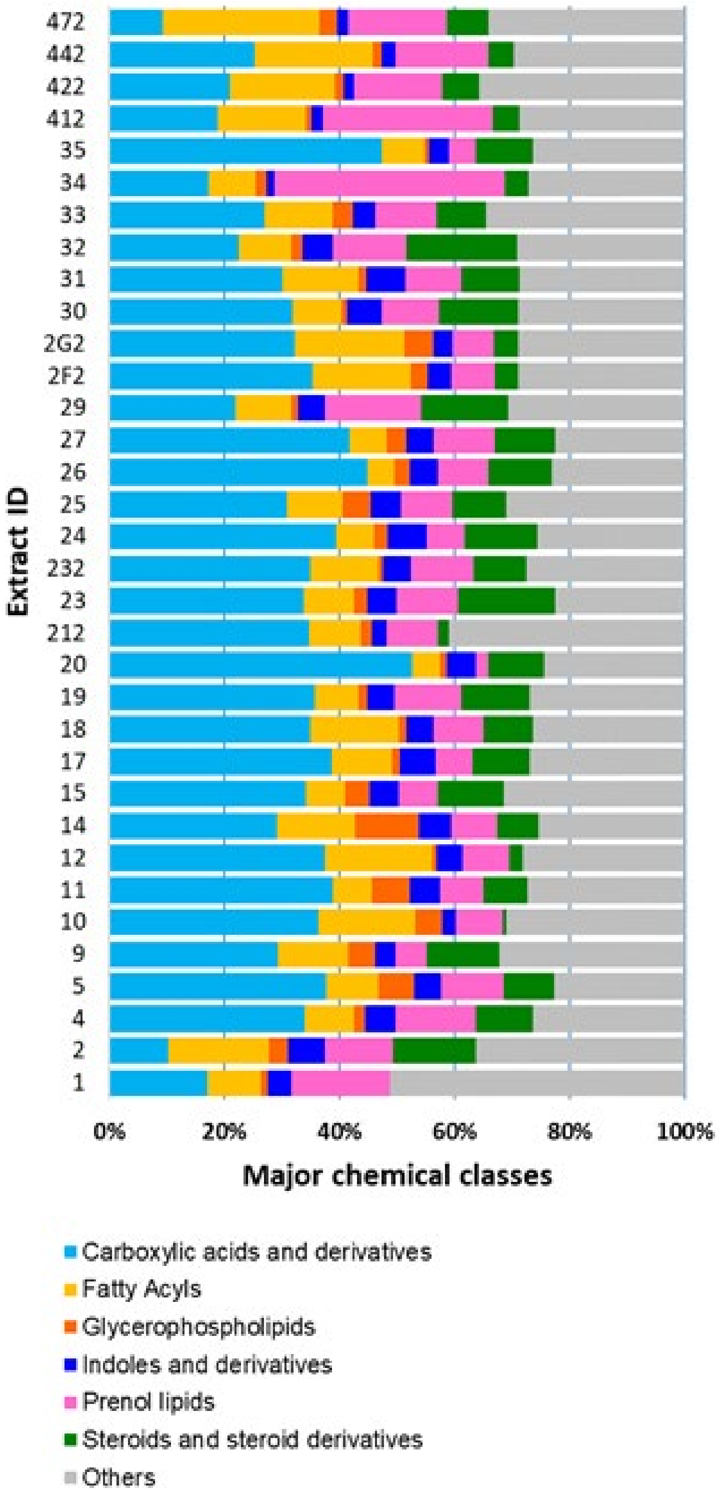

Figure S4. Chemical richness per bacteria strain. **Dereplicated** metabolites were grouped according to ClassyFire chemical taxonomy. The best-represented chemical classes in the whole study are carboxylic acids and derivatives, fatty acid-derived compounds, prenol lipids and steroids and derivatives; glycerophospholipides and indole and derivatives are also well represented. The bar plot indicates the percentage of annotated features belonging to a given chemical class per each bacteria extract.

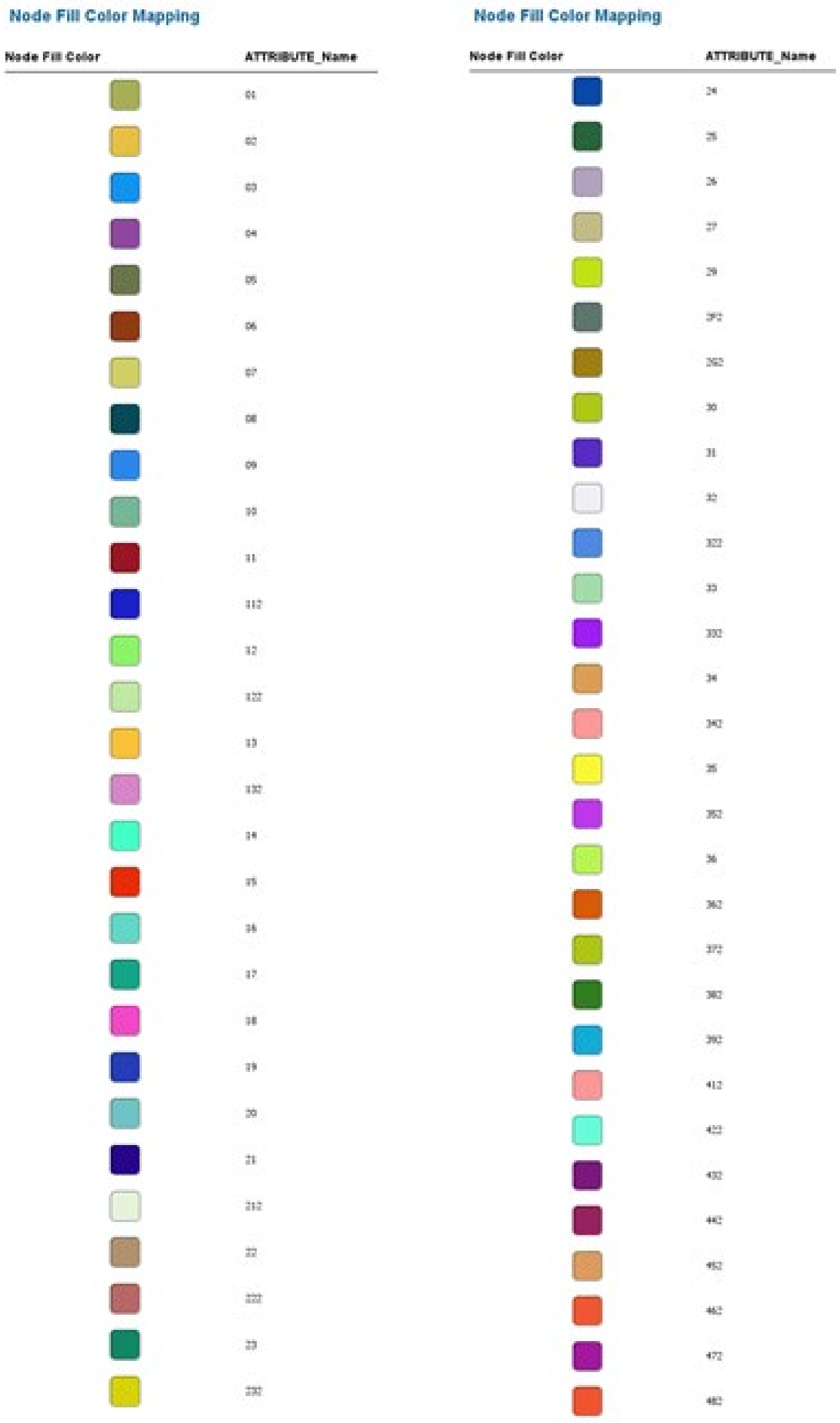

Figure S5. Color-code legend for Figure 4A. **Node** filling color mapping per bacteria strain ("ATTRIBUTE_Name"); strain 312 is not represented because it has no strain-specific features.

Supplementary Tables

Table S1. Composition of media and solutions used for isolation and cultivation of bacterial strains**.**

DSMZ medium 1649 Artificial Sea Water (ASW) salts -HD (1:10 diluted)

Compound	Amount
Pepton	0.5 g
Glucose (D)	0.1 g
Yeast Extract (H)	0.25 g
ASW[a]	1000.00 mL
Adjust pH to 7.3	
Add to 1000 mL of medium after autoclaving:	
Trace element solution SL-10[b]	1.00 mL
Vitamin solution[c]	1.00 mL

DSMZ medium 1649 Artificial Sea Water (ASW) salts -HD (1:10 diluted) Polymer

Compound	Amount
Peptine	1.0 g
Chitin	1.0 g
Cellulose	1.0 g
Xylan	1.0 g
Curdlan	1.0 g
ASW[a]	1000.00 mL
Adjust pH to 7.3	
Add to 1000 mL of medium after autoclaving:	
Trace element solution[b]	1.00 mL
Vitamin solution[c]	1.00 mL

KM14

Compound	Amount
Acetate	0.05 g
Meat Extract	0.04 g
Peptone	0.04 g
Yeast extract	0.02 g
Casamino acids	0.06 g
Sucrose	0.03 g
Soluble starch	0.03 g
Cellulose	0.01 g
Urea	0.005 g
NH_4Cl	0.057 g
NH_4HCO_3	0.06 g
KH_2PO_4	0.034 g
$NaHCO_3$	0.394 g
$CaCl_2$ x $2H_2O$	0.22 g
$MgSO_4$ x $7H_2O$	0.15 g
EDTA	0.0003 g
Trace element solution[b]	1ml
ABW14[a]	1000.00 mL

Medium "insoluble humic analogs"

Compound	Amount
Abietic acid	500 µM
Quercetin	500 µM
Coumestrol	500 µM
Methyl cinnamate	500 µM

ASW[a]	1000.00 mL
Adjust pH to 7.3	
Add to 1000 mL of medium after autoclaving:	
Trace element solution SL-10[b]	1.00 mL
Vitamin solution[c]	1.00 mL

Medium "soluble humic analogs"

Compound	Amount
Salicylate	500 μM
Phtalic acid	500 μM
AQDS	500 μM
Furfural	500 μM
Hydroxymethylfurfural	500 μM
Lignosulfonate	500 μM
Basal medium[a]	1000.00 mL
Adjust pH to 7.3	
Add to 1000 mL of medium after autoclaving:	
Trace element solution SL-10[b]	1.00 mL
Vitamin solution[c]	1.00 mL

DSMZ medium 1426 Soil Solution Equivalent (SSE)/HD 1:10

Compound	Amount
Peptone	0.50 g
Yeast extract	0.25 g
Glucose	0.10 g
4-(2-hydroxyethyl)-1-piperazineethanesulfonic acid (HEPES)	10 mM
Soil Solution Extract medium[d]	500 mL
Distilled Water	500 mL
Add to 1000 mL of medium after autoclaving:	
Trace element solution SL-10[b]	1.00 mL
Vitamin solution[c]	1.00 mL

Medium Soil Solution Equivalent SSE/HP

Compound	Amount
Pepton	0.10 g
Yeast extract	0.10 g
4-(2-hydroxyethyl)-1-piperazineethanesulfonic acid (HEPES)	10 mM
Soil Solution Extract medium[d]	500 mL
Distilled Water	500 mL
Add to 1000 mL of medium after autoclaving:	
Trace element solution SL-10[b]	1.00 mL
Vitamin solution[c]	1.00 mL

DSMZ medium 514 Medium BACTO MARINE BROTH (MB, DIFCO 2216)

Compound	Amount
Bacto peptone	5.00 g
Bacto yeast extract	1.00 g
Fe(III)citrate	0.10 g
NaCl	19.45 g
$MgCl_2$	5.90 g
Na_2SO_4	3.24 g
$CaCl_2$	1.80 g
KCl	0.55 g
$NaHCO_3$	0.16 g
KBr	0.08 g

$SrCl_2$	34.00 mg
H_3BO_3	22.00 mg
Na-silicate	4.00 mg
NaF	2.40 mg
$(NH_4)NO_3$	1.60 mg
Na_2HPO_4	8.00 mg
Distilled water	1000.00 ml
$Na_2S_2O_3$ x $5H_2O$	1.00 g/l
Final pH should be 7.6 ± 0.2 at 25°C. If using the complete medium from Difco add 37.40 g to 1 litre water.	

L1ZM10

Compound	Amount
L1 medium (https://www.ccap.ac.uk/index.php/media-recipes) amended with 0.05% (w/v) peptone , 0.01% (w/v) yeast extract and 1.5% (w/v) agar.	
Peptone	0.5 g
Yeast extract	0.1 g
Agar	15 g
Filtered natural sea water	1000.00 mL

Solutions

Depending on the salinity of the seawater samples, media were based either on artificial sea water media (ASW[a], modified from Bruns et al., 2003) or artificial brackish water (ABW14[a])

[a]Artificial Sea Water media (ASW[a]; modified from Bruns et al., 2003)

Compound	Amount
NaCl	23.6 g
$MgCl_2 \cdot 7H_2O$	4.53 g
$CaCl_2 \cdot 2H_2O$	1.3 g
KCl	0.64 g
$MgSO_4 \cdot 7H_2O$	5.94 g
Na_2HPO_4	0.01 g
NH_4NO_3	2.1 mg
4-(2-hydroxyethyl)-1-piperazineethanesulfonic acid (HEPES)	2.3 g
Distilled water	1000.00 mL

[a]Artificial Brackish Water (ABW14[a]) salinity 14 PSU

Compound	Amount
NaCl	7.7483 g
$MgCl_2 \cdot 6H_2O$	3.87086 g
$CaCl_2 \cdot 2H_2O$	0.47334 g
KCl	0.20874 g
Na_2SO_4	1.27274 g
$NaHCO_3$	0.35952 g
KBr	0.03199 mg
125x salt solution	8 ml
HEPES	2.38g
Distilled water	1000.00 mL

[b]Trace element solution SL10

Compound	Amount
HCl (25%; 7.7 M)	10 mL
$FeCl_2$ x $4H_2O$	1.50 g
$ZnCl_2$	70.0 mg
$MnCl_2$ x $4H_2O$	100.0 mg

H_3BO_3	6.0 mg
$CoCl_2$ x $6H_2O$	190.0 mg
$CuCl_2$ x $2H_2O$	2.0 mg
$NiCl_2$ x $6H_2O$	24.0 mg
Na_2MoO_4 x $2H_2O$	36.0 mg
Distilled water	990 mL
First dissolve $FeCl_2$ in HCl, then dilute with water, add and dissolve the other salts. Finally make up to 1000 mL.	

[c]Vitamin solutions

Compound	Amount
Biotin	2.0 mg
Folic acid	2.0 mg
Pyridoxine-HCl	10.0 mg
Thiamine-HCl x $2H_2O$	5.0 mg
Riboflavin	5.0 mg
Nicotinic acid	5.0 mg
D-Ca-pantothenate	5.0 mg
Vitamin B_{12}	0.10 mg
p-aminobenzoic acid	5.0 mg
Lipoic acid	5.0 mg
Distilled	1000 mL

[d]Soil Solution Equivalent medium (SSE, double concentrated)

Compound	Amount
$CaCl_2$ x $2H_2O$ 0.2938 g	0.2938 g
NH_4Cl	0.1069 g
$MgCl_2$ x $6H_2O$	0.2036 g
$(NH_4)_2SO_4$	0.1983 g
$MgSO_4$ x $7H_2O$	0.7390 g
$CaSO_4$ x $2H_2O$	0.8606 g
$Ca(NO_3)_2$ x $4H_2O$	0.2360 g
$NaNO_3$	0.4240 g
KH_2PO_4	0.5000 ml
$FeSO_4$ x $7H_2O$	0.0111 g
K_2SO_4	0.0870 g
Distilled water	1000 ml
Dissolve by shaking overnight. Sterilize by autoclaving.	

[e]Chemoattractants used in the chemotaxis experiments

Compound	Amount
Tween	0.001%
DMSO	1%
Mix sugars I (trehalose, cellobiose, maltose)	2 mM each
Mix sugars II (gentobiose, sucrose)	2 mM each
Mix sugars III (N-acetylglucosamine, mannitol, rhamanose)	2 mM each
KH_2PO_4	2 mM each
20 Amino acids	2 mM each
Fatty Acid mix (formate, acetate, valerate, propionate, butyrate)	2 mM each
TCA mix (lactate, succinate, citrate, pyruvate, oxaloacetate, α-ketoglutarate)	2 mM each
Nitrogen compounds (NH_4^+, TMAO, urea)	1 mM each

[f]Salt Solution (1000x)

Compound	Amount
H_3BO_3	0.79646 g

$SrCl_2$	0.68894 g
NH_4Cl	1.2691112 g
KH_2PO_4	0.094052 mg
NaF	0.1715 mg
Distilled water	100.00 mL

Table S2. Data processing parameters. Pre-processing parameters setting for generating the feature table in MZmine2 and exporting the .mgf and quantification table to be used in GNPS and SIRIUS4.

**Parameter**	**Value**
Mass detection	
MS1	1.5E3
MS2	1.5E2
ADAP Chrom. building	
group int.	1.5E3
min ht	4.5E3
tolerance (ppm)	20
Deconvolution (baseline cutoff)	
min peak ht	4.5E3
peak dur. range (min)	10
baseline lev.	1.5E3
m/z range for MS2 pairing (Da)	0.01
RT range for MS2 pairing (min)	0.2
Isotopic peak grouper	
m/z tolerance (ppm)	20
RT tolerance (min)	0.8
Joint aligner	
m/z tolerance (ppm)	20 (wt 75)
RT tolerance (min)	0.8 (wt 25)
Duplicate peak filter	
m/z tolerance (ppm)	10
RT tolerance (min)	0.5
Peak row filtering	
RT (min)	0.9-21
MS2 only	
IIMN	
row grouping (metacorr)	
RT tolerance (min)	0.8
min ht	1.5E3
noise level	1.5E2
IIN	M+H, M+Na, M+K, M+NH4, 2M+H
m/z tolerance (ppm)	15
min ht	0

Table S3. Features additionally dereplicated with the present workflow.

m/z	r.t. (min)	Compound name	Conf. level	CluMSID cluster # [1]	Other molecules present in the cluster
**160.0759**	**7.2**	Indole-3-acetaldehyde	2	33	Tryptophan, Nortriptyline ISTD
**162.0759**	**1.4**	5-Methyl DL-Glutamate	3	4	Glutamate, N-acetylglutamate, Glutathion disulfide
**174.0552**	**4.6**	Quinoline-2-Carboxylic Acid	3	14	Anthranilate, 2-aminophenol
**176.0704**	**7.8**	Methyl indole-3-carboxylate	3	10	HHQ fragment
**194.0674**	**5.8**	2-Amino-3-methyl-8H-pteridine-4,7-dione	3	0	Pterine and others
**195.1133**	**6.3**	Proline anhydride	3	6	Proline, Tyrosine, Phenylalanine, peptides
**197.1288**	**7.0**	Cyclo(Pro-Val)	3	6	Proline, Tyrosine, Phenylalanine, peptides
**202.1221**	**9.8**	8-methyl-2-propyl-4-quinolinol	3	43	Alkyl quinolones
**257.1036**	**7.4**	N-(2,6-Dimethylphenyl)-2-Pyrrolidinecarboxamide	3	6	Proline, Tyrosine, Phenylalanine, peptides
**261.1244**	**6.9**	Cyclo(L-Pro-L-Tyr)	3	6	Proline, Tyrosine, Phenylalanine, peptides
**317.1452**	**1.8**	N-Acetylglutaminylglutamine	3	4	Glutamate, N-acetylglutamate, Glutathion disulfide
**321.1016**	**12.4**	5-Methyl-N-(4-Sulfamoyl phenethyl)Pyrazine-2-Carboxamide	3	23	Glipizide ISTD
**322.1070**	**2.2**	S-Methylglutathione	3	4	Glutamate, N-acetylglutamate, Glutathion disulfide
**323.0705**	**5.1**	Gamma-Glutamyl-S-Allylmercaptocysteine	3	4	Glutamate, N-acetylglutamate, Glutathion disulfide
**326.2082**	**7.0**	1-(1-L-Leucyl-L-prolyl)-L-proline	3	6	Proline, Tyrosine, Phenylalanine, peptides
**338.3421**	**22.3**	N-Pentadecyl cyclohexanecarboxamide	3	63	Rhamnolipids (putative)
**359.2799**	**16.0**	3-Hydroxydecanoyl-3-Hydroxydecanoate	3	21	Rhamnolipids (putative)
**359.2799**	**17.0**	1,3-Dioctanoyl-1,2,3-Butanetriol	3	63	Rhamnolipids (putative)
**387.3113**	**18.5**	[(2S)-3-hydroxy-2-pentanoyloxypropyl] tetradecanoate	3	63	Rhamnolipids (putative)
**387.3114**	**17.5**	Dipropylene Glycol Dicaprylate	3	63	Rhamnolipids (putative)
**415.3422**	**18.9**	(2s)-3-Hydroxy-2-(Nonanoyloxy)Propyl Laurate	3	63	Rhamnolipids (putative)
**452.2773**	**14.7**	LPE(16:1/0:0)	3	26	PG(16:1/0:0)
**522.3556**	**17.4**	PC(18:1/0:0)	3	6	PC(16:0/0:0) (putative) and Proline, Tyrosine, Phenylalanine, peptides
**891.3632**	**12.4**	Glipizide (2M+H)	2	23	Glipizide ISTD

[1] T. Depke, R. Franke, M. Brönstrup, *J. Chromatogr. B Anal. Technol. Biomed. Life Sci.* **2017**, *1071*, 19–28.

Supplementary Text

Validation of annotation procedure

The dereplication workflow utilized and described in the main text was validated by comparing the dereplication obtained with the present approach to a method previously developed in our group,[1] using the same biological sample and dataset of a Pseudomonas aeruginosa PA14 cell extract.
In particular, we processed the .mzXML file generated from the LC-MS measurement of *Pseudomonas aeruginosa* PA14, extracted with 80% (v/v) methanol and analysed by positive mode electrospray ionization quadrupole time-of-flight mass spectrometry in full scan mode on the same instrument used for the present investigation (maXis™ HD QTOF, Bruker, Bremen, Germany).
Subsequent data analysis steps were carried out exactly as done for the present data set and as described in the Methods section. 603 features were defined by this approach *vs* 518 consensus spectra obtained from R processing steps described in the paper from our colleagues Depke et al.[1] (493 features were common to both processing approaches and were considered to compare the annotation).
To evaluate the annotation accuracy of our approach, we ran the dereplication first by matching them with analytical standards present in our in-house library, then by matching MS/MS spectra with externally acquired molecular structures present in online databases such as GNPS; finally by calculating their accurate masses, isotopic distribution patterns and fragmentation trees with the in silico structure prediction software SIRIUS4, coupled with CSI:FingerID.
Our method annotated 104 metabolites of the 493 "common" features compared to what previously published by Depke et al.[1], who annotated 125. Among these 104 dereplicated features, 80 correspond to the identification published by Depke, while 24 were dereplicated only by our workflow, but not reported by Depke. The identity of these 24 additional features (confidence level 2 or 3) was checked, and confirmed in all cases through a manual re-analysis. These molecules are presented in the Table S3.
The annotation method of Depke et al.[1] was partially manual and included a semi-targeted analysis of alkyl quinolones. This approach allowed to putatively annotate additional 31 metabolites, including alkyl quinolones and rhamnolipids, that we could not annotate with our method. Additional 14 fragments of metabolites were annotated in the paper by Depke, thanks to a manual approach, but they could not be dereplicated with the workflow presented here.
Such validation experiment demonstrated the efficacy of the dereplication approach described in this work, which is comparable to a semi-targeted and manual curated method.
